# Double-bond-containing polyallene-based triblock copolymers via phenoxyallene and (meth)acrylate

**DOI:** 10.1038/srep43706

**Published:** 2017-03-02

**Authors:** Aishun Ding, Guolin Lu, Hao Guo, Xiaoyu Huang

**Affiliations:** 1 Department of Chemistry, Fudan University, 220 Handan Road, Shanghai 200433, People’s Republic of China; 2 Key Laboratory of Synthetic and Self-Assembly Chemistry for Organic Functional Molecules, Shanghai Institute of Organic Chemistry, Chinese Academy of Sciences, 345 Lingling Road, Shanghai 200032, People’s Republic of China

## Abstract

A series of ABA triblock copolymers, consisting of double-bond-containing poly(phenoxyallene) (PPOA), poly(methyl methacrylate) (PMMA), or poly(butyl acrylate) (PBA) segments, were synthesized by sequential free radical polymerization and atom transfer radical polymerization (ATRP). A new bifunctional initiator bearing azo and halogen-containing ATRP initiating groups was first prepared followed by initiating conventional free radical homopolymerization of phenoxyallene with cumulated double bond to give a PPOA-based macroinitiator with ATRP initiating groups at both ends. Next, PMMA-*b*-PPOA-*b*-PMMA and PBA-*b*-PPOA-*b*-PBA triblock copolymers were synthesized by ATRP of methyl methacrylate and *n*-butyl acrylate initiated by the PPOA-based macroinitiator through the site transformation strategy. These double-bond-containing triblock copolymers are stable under UV irradiation and free radical circumstances.

The presence of any unique polymeric structure will definitely influence the properties of polymer since the nature of polymer rests with its structure. Polyallene, which can be obtained by the selective polymerization of either part of cumulated double bonds of allene derivatives, is expected to provide many idiographic properties because of its unique structure of possessing reactive exomethylene or substituted exomethylene directly linked to the backbone. This kind of reactive polymer has been intensively investigated because they can be utilized as attractive synthetic precursors for functional materials due to the versatility of addition reactions of double bonds^1^.

Allene derivatives are often regarded as the isomers of propargyl derivatives since they possess cumulated double bonds and they can be polymerized by many different approaches such as free radical, cationic, coordination, and zwitterionic polymerizations^2^. In particular, Endo *et al*. developed Ni-catalyzed living coordination polymerization of allene derivatives to provide well-defined polymers with controlled molecular weights and narrow molecular weight distributions in high yields under mild conditions using [(*η*^3^-allyl)NiOCOCF_3_]_2_ as initiator^3^,^4^,^5^,^6^,^7^,^8^,^9^. Allene derivatives can also be block copolymerized since that living π-allylnickel end groups are stable and the molecular weights can be adjusted by the feeding ratio^7^,^10^,^11^. Furthermore, block copolymers of allene derivatives with butadiene^12^, isocyanides^13^, or 3-hexylthiophene^14^,^15^ have been synthesized by using two stages of monomer feeding because all these monomers can be polymerized with the same π-allylnickel catalyst.

However, facile preparation of block copolymers from allene derivatives and common vinyl monomers including styrene and (meth)acrylate still remains a challenge because the polymerizations of usual vinyl monomers can not be initiated by [(*η*^3^-ally)NiOCOCF_3_]_2_. Although random radical copolymerization of allene derivatives and common vinyl monomers could easily introduce the reactive double bonds into the conventional polymers, the molecular weights of the obtained copolymers are very difficult to be controlled and the molecular weight distributions are generally broad^16^. Moreover, living radical polymerization such as atom transfer radical polymerization (ATRP), single-electron-transfer living radical polymerization (SET-LRP), and reversible addition-fragmentation chain transfer (RAFT) polymerization of allene derivatives has not been reported until now, which may be attributed to the unusual cumulated double bonds. Therefore, the applications of polyallene as reactive polymers have been certainly limited. In order to enlarge the application range of polyallene, it is necessary to prepare tailor-made polymeric architectures containing polyallene segment. To realize this goal, block copolymer with a stable covalent-bonded linkage between two different segments is a good and convenient choice. The interest in block copolymers arises mainly from their unique solution and associative properties as a consequence of their molecular structure. To our best knowledge, none has reported the block copolymer via allene derivative and common vinyl monomer until now.

Generally, two strategies have been employed to synthesize the block copolymers: one is the sequential feeding of different monomers through living polymerization, including anionic^17^,^18^,^19^, cationic^20^,^21^, group transfer^22^, living radical polymerization^23^,^24^,^25^,^26^; on the other hand, the site transformation strategy was used to synthesize the block copolymers via different polymerization mechanisms^27^. Thus, bi- and multi-functional initiators have been designed and prepared in order to satisfy different polymerization mechanism demands of the monomers with different chemical structures^28^,^29^,^30^,^31^,^32^,^33^. By this way, end-functionalized macroinitiator was first prepared and subsequently used to initiate the polymerization of the second monomer by another polymerization mechanism. In principle, any block copolymer difficult to be prepared by single polymerization mechanism could be designed and synthesized by this approach.

Recently, metal-catalyzed living radical polymerization including ATRP^34^,^35^,^36^,^37^,^38^,^39^ and SET-LRP^40^,^41^,^42^,^43^,^44^,^45^ has been widely used for the synthesis of polymers with various structures, especially for the synthesis of block polymers. However, ATRP still does not work for some monomers though it has many advantages compared to other polymerization methods when used for block copolymerization. An alternative method to overcome this limitation is to combine ATRP with conventional radical polymerization by using bifunctional initiator.

In light of those aforementioned considerations, a new bifunctional initiator containing azo and ATRP initiating groups was first synthesized in the present work, which could sequentially initiate the polymerizations of allene derivative and commonly used vinyl monomer under different conditions as shown in Fig. 1. The free radical method is an easiest way to realize the polymerization of allene derivative, while ATRP is an attractive approach for common vinyl monomer. In this work, we reported the first example of triblock copolymers containing phenoxyallene and (meth)acrylate repeated units prepared by a combination of conventional azo-initiated free radical polymerization and ATRP, which showed high stability under free radical and UV irradiation surroundings.

## Results and Discussion

### Synthesis and characterization of azo-ATRP bifunctional initiator

As mentioned above, it was difficult to prepare block copolymers of allene derivatives and vinyl monomers by single polymerization mechanism, i.e. sequential feeding, because nickel-catalyzed living coordination polymerization is only suitable for allene derivatives^3^,^4^,^5^,^6^,^7^,^8^,^9^, diene^12^, isocyanide^13^, and thiophene^14^,^15^, not suitable for common vinyl monomers. Therefore, the “site transformation” strategy is the only feasible approach to construct block copolymers of allene derivatives and vinyl monomers, in which end-functionalized polyallene can be employed as macromolecular initiator or mediation agent to initiate or mediate the polymerization of the second monomer with diverse polymerization techniques such as ATRP^34^,^35^,^36^, SET-LRP^40^,^41^, and RAFT polymerization^46^,^47^. ATRP appears to be the most popular process among different reversible-deactivation radical polymerization (RDRP) techniques because of its mild reactions conditions, tolerance of monomer functionalities, and variety of monomers, and it has been extensively used to prepare copolymers with different architectures, making it not difficult to synthesize polyallene-based block copolymers. Indeed, our group has reported the synthesis of well-defined polyallene-based graft copolymers via the combination of Ni-catalyzed living coordination polymerization and ATRP^48^,^49^,^50^.

**Figure 1:**
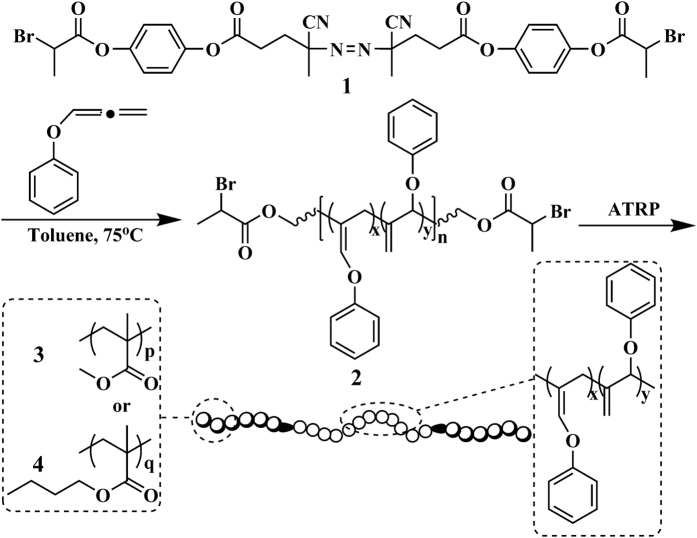
Synthesis of polyallene-based triblock copolymer.

It has been reported that allene derivatives could be easily polymerized via common free radical polymerization using 2,2′-azobis(isobutyronitrile) (AIBN) as initiator^2^, thus we firstly designed a bifunctional initiator **1** comprising one azo group at the center and two halogen-containing ATRP initiating groups at both ends as shown in Fig. 1. This compound was synthesized using 2-bromopropionyl chloride, hydroquinone, and 4,4′-azobis(4-cyanopentanoic acid) as starting materials (see Supporting Information). 4,4′-Azobis(4-cyanopentanoic acid) was first quantitatively transformed into an intermediate of 4,4′-azobis(4-cyanopentanyl chloride) by reacting with thionyl chloride; meanwhile, 2-bromopropionyl chloride was treated with hydroquinone to give another intermediate of 4-hydroxyphenyl 2-bromopropanoate. Both intermediates reacted at room temperature to afford the desired azo-ATRP bifunctional initiator **1**.

**Figure 2:**
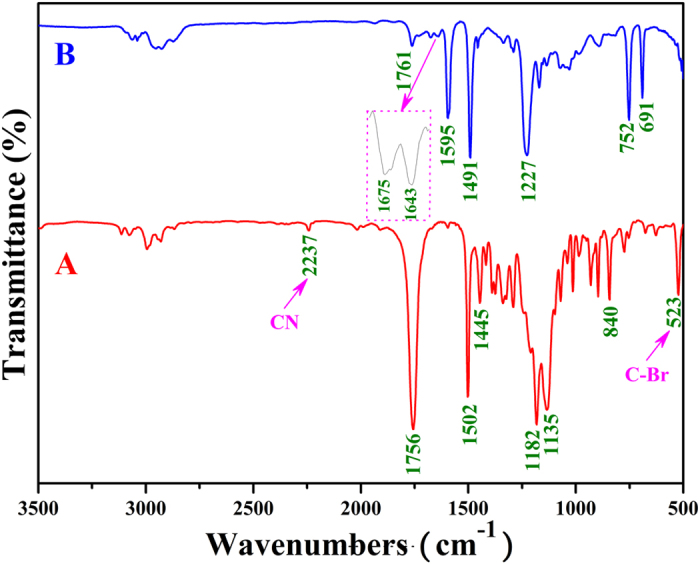
FT-IR spectra of bifunctional initiator **1** (**A**) and Br-PPOA-Br **2** (**B**).

**Figure 3:**
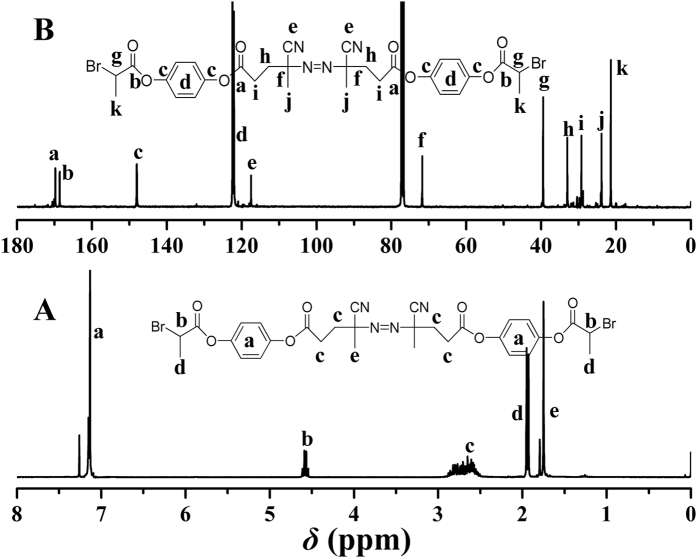
^1^H (**A**) and ^13^C (**B**) NMR spectra of bifunctional initiator **1** in CDCl_3_.

The chemical structure of bifunctional initiator **1** was examined by FT-IR, ^1^H NMR, ^13^C NMR, and elemental analysis in detail. As shown in Fig. 2A, the IR absorptions at 2237 and 523 cm^−1^ denoted the presence of -C≡N group and C-Br bond, respectively, while the stretching vibration of carbonyl appeared at 1756 cm^−1^. Figure 3A shows ^1^H NMR spectrum of bifunctional initiator **1**, which exhibited all typical proton resonance signals related with azo and ATRP initiating groups. The peaks at 4.59 (‘b’, 1 proton) and 1.95 (‘d’, 3 protons) ppm were attributed to 4 protons of O_2_CC*H*(C*H*_3_)Br initiating group. The resonance signals located at 2.61 (‘c’, 4 protons) and 1.75 (‘e’, 3 protons) ppm corresponded to 7 protons of C*H*_3_C(CN)C*H*_2_C*H*_2_CO_2_ adjacent to azo group. The distinct carbon resonance signals appearing at 21.4 (‘k’) and 39.4 (‘g’) ppm in ^13^C NMR spectrum (Fig. 3B) further proved the existence of Br-containing ATRP initiating group. Moreover, the elemental analysis result was well consistent with the theoretical value. Thus, all aforementioned results evidenced the successful synthesis of bifunctional initiator **1**, which can combine free radical polymerization with ATRP to prepare the block copolymers unavailable via single polymerization mechanism.

### Synthesis and characterization of Br-PPOA-Br macroinitiator

Since azo group may induce the chain transfer of propagating radicals during ATRP, conventional free radical polymerization of phenoxyallene (POA) initiated by bifunctional initiator **1** should be firstly conducted. Therefore, solution free radical polymerization of POA was performed in toluene at 75 °C under Ar using the as-prepared **1** as initiator in order to obtain Br-PPOA-Br **2** macroinitiator.

The resulting homopolymer was characterized by GPC, FT-IR, ^1^H NMR, ^13^C NMR, and elemental analysis. The homopolymer showed a unimodal elution peak in GPC retention curve with a broad molecular weight distribution of 1.95, which was the characteristic of free radical polymerization. The peaks at 1643 and 1675 cm^−1^ in FT-IR spectrum after homopolymerization of POA (Fig. 1B) corresponded to the double bonds originating from the cumulated double bonds of allene derivatives. The stretching vibration peak of carbonyl appeared at 1761 cm^−1^, this indicating the presence of ATRP initiation group in PPOA homopolymer.

**Figure 4:**
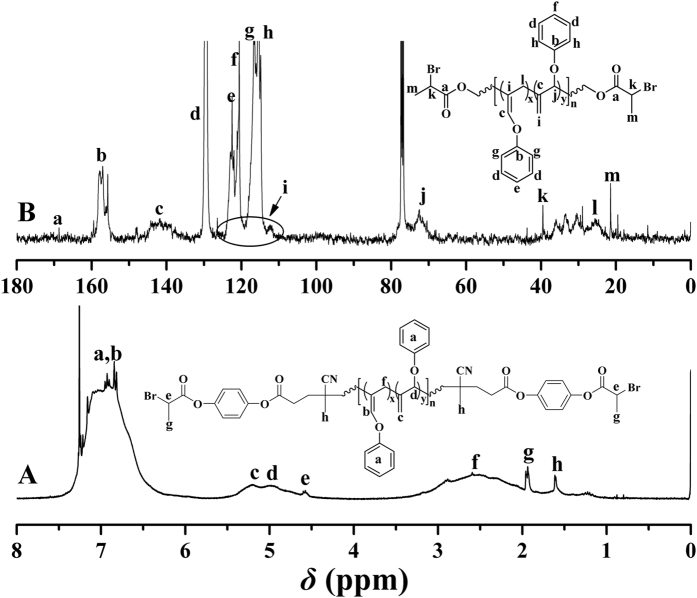
^1^H (**A**) and ^13^C (**B**) NMR spectra of Br-PPOA-Br **2** in CDCl_3_.

Figure 4A shows ^1^H NMR spectrum of the homopolymer, which displayed that PPOA homopolymer comprised both 1,2- and 2,3-polymerized units (labeled as y and x in Fig. 1). PPOA homopolymer contained 28% of 1,2-polymerized units and 72% of 2,3-polymerized units, i.e. less 1,2-polymerized units due to the steric hindrance, via the integration area ratio of 1,2-polymerized units (peak ‘d’ at 5.00 ppm) to 2,3-polymerized units (peak ‘f’ at 2.55 ppm). The resonance signals at 1.61 ppm (“h”) belonged to 3 protons of C*H*_3_CCN previously adjacent to azo group in bifunctional initiator **1**, which clearly demonstrated the polymerization of POA was indeed initiated by the azo group in compound **1**. The signals originating from 4 protons of O_2_CC*H*(C*H*_3_)Br initiating group were located at 4.57 (‘e’, 1 proton) and 1.93 (‘g’, 3 protons), respectively, which illustrated that the halogen-containing ATRP initiating group was inert during the free radical polymerization of POA. Figure 4B shows ^13^C NMR spectrum of the homopolymer and the carbon resonance signal of the double bond was found to be located at 141.8 ppm (‘c’) while another signal was overlapped with the signal of phenyl. In addition, the carbon resonance signals of O_2_*CC*H(*C*H_3_)Br initiating group appeared at 21.3 (‘m’, *C*H_3_), 39.4 (‘k’, *C*HBr), and 169.0 (‘a’, *C*O_2_) ppm, respectively. Thus, we can confirm the formation of PPOA homopolymer containing terminal ATRP initiating group.

Though polystyrene-calibrated GPC provided a relative *M*_n_ of 9,400 g/mol, the ‘absolute’ molecular weight (*M*_n,GPC/MALS_) was determined by GPC/MALS with a value of 9,200 g/mol, which was very close with that obtained from conventional GPC because the structure of PPOA was similar with that of polystyrene. Generally, the termination of free radical polymerization includes coupling and disproportionation and the ratio between both mechanisms can be determined by measuring the exact number of characteristic end group. Herein, if there was only coupling termination, the number of O_2_CCH(CH_3_)Br end group should be 2.0; if there was only disproportionation termination, the number of O_2_CCH(CH_3_)Br end group should be 1.0; if there was both coupling and disproportionation termination, the number of O_2_CCH(CH_3_)Br end group should be between 1.0 and 2.0. Given that PPOA homopolymer possessed two O_2_CCH(CH_3_)Br initiating groups at both ends, the bromine content (Br%) should be 1.74% ( = 160/9,200). In the current case, elemental analysis afforded a Br% of 1.72% for PPOA homopolymer, which was very close to the theoretical value (1.74%). This fact strongly verified that PPOA homopolymer possessed two O_2_CCH(CH_3_)Br initiating groups at both ends, i.e. Br-PPOA-Br **2** macroinitiator. Besides, the macroinitiator can dissolve in common organic solvents including THF, CHCl_3_, toluene, and DMF, which is convenient for the subsequent ATRP; but it can not dissolve in *n*-hexane, methanol, and water.

### Synthesis of PMMA-*b*-PPOA-*b*-PMMA triblock copolymer

PMMA-*b*-PPOA-*b*-PMMA **3** triblock copolymer was constructed via bulk ATRP of methyl methacrylate (MMA) initiated by Br-PPOA-Br **2** using CuBr/dHbpy as catalytic system at 50 °C (Table 1). All resulting products possessed higher molecular weights compared to that of Br-PPOA-Br **2** macroinitiator, which proved the occurrence of the polymerization of MMA monomer. The molecular weights of products ascended with longer polymerization time, a feature of ATRP^51^.

**Figure 5:**
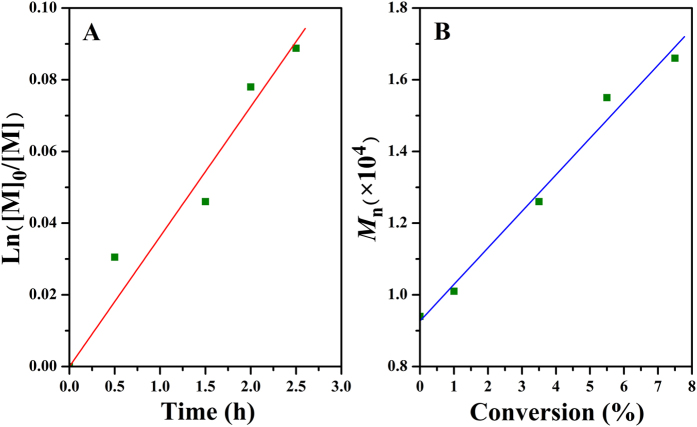
(**A**) Kinetic plot for bulk ATRP of MMA at 50 °C initiated by Br-PPOA-Br **2** and (**B**) dependence of *M*_n_ and *M*_w_/*M*_n_ on conversion of MMA.

Polymerization kinetics of bulk ATRP of MMA was investigated by measuring the conversion of MMA via GC and the molecular weight of the product via GPC. The semilogarithmic plot of Ln([M]_0_/[M]) *vs*. time is depicted in Fig. 5A, which shows that the conversion of MMA increased linearly with the extending of polymerization time. This first order polymerization kinetics clearly showed a constant number of propagating species during the polymerization, which is a typical character of ATRP^51^. Furthermore, the molecular weight of the obtained polymers linearly increased with the rising of the conversion of MMA as shown in Fig. 5B, which was also identical with the feature of ATRP^51^.

**Figure 6:**
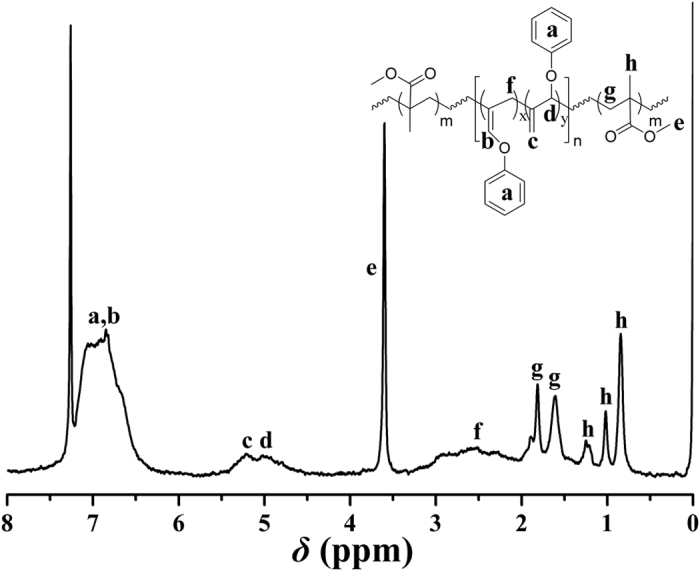
^1^H NMR spectrum of PMMA-*b*-PPOA-*b*-PMMA **3** in CDCl_3_.

The obtained block copolymer was examined by ^1^H NMR and all typical proton resonance signals of PPOA and PMMA segments could be found in Fig. 6. The characteristic signals of PMMA segments appeared at 0.84, 1.02, 1.23 (‘h’), and 3.60 (‘e’) ppm, corresponding to the protons of CH_2_CC*H*_3_ and CO_2_C*H*_3_ of MMA repeated unit, respectively. The signals between 4.50 ppm and 5.50 ppm attributed to the protons of the double bonds in POA repeated unit evidenced that the double bonds of PPOA block kept inert during ATRP of MMA. Thus, it can be concluded from the above-mentioned results that double-bond-containing PMMA-*b*-PPOA-*b*-PMMA **3** triblock copolymers were successfully synthesized through ATRP of MMA initiated by Br-PPOA-Br **2** macroinitiator.

### Synthesis of PBA-*b*-PPOA-*b*-PBA triblock copolymer

Similarly, bulk ATRP of *n*-butyl acrylate (BA) was conducted at 80 °C using Br-PPOA-Br **2** as macroinitiator and CuBr/PMDETA as catalytic system. It can be seen from Table 2 that all three resultants possessed higher molecular weights in comparison with that of Br-PPOA-Br **2** macroinitiator, this indicating the performance of ATRP of BA. Moreover, it was also found that the extension of polymerization time led to the raising of the molecular weights of the resulting products, this according with the characteristic of ATRP^51^.

**Figure 7:**
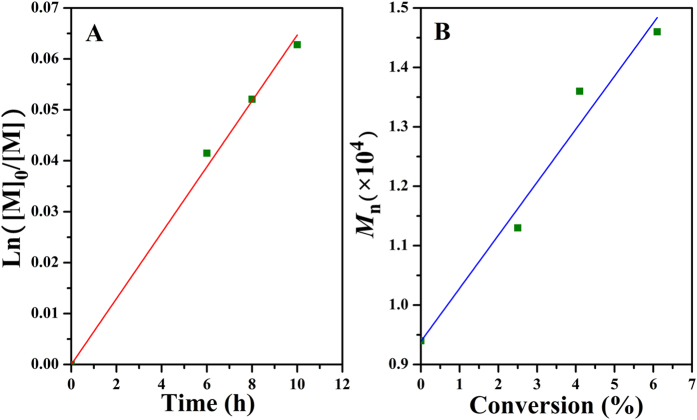
(**A**) Kinetic plot for bulk ATRP of BA at 80 °C initiated by Br-PPOA-Br **2** and (**B**) dependence of *M*_n_ and *M*_w_/*M*_n_ on conversion of BA.

The conversions of BA monomer and the molecular weights of the resultants were determined by GC and GPC, respectively, so as to study the polymerization kinetics of bulk ATRP of BA. Figure 7A shows the semilogarithmic plot of Ln([M]_0_/[M]) *vs*. time, which exhibited the linear dependence of the conversion of BA on the extending of polymerization time, i.e. first order polymerization kinetics. This point distinctly demonstrated the typical character of ATRP^51^ with a constant number of propagating species during the polymerization. In addition, the evolution of the molecular weight of the obtained polymers is shown in Fig. 7B and it is obvious that the molecular weight is in first order with the conversion of BA monomer, which is consistent with the characteristic of ATRP^51^.

**Figure 8:**
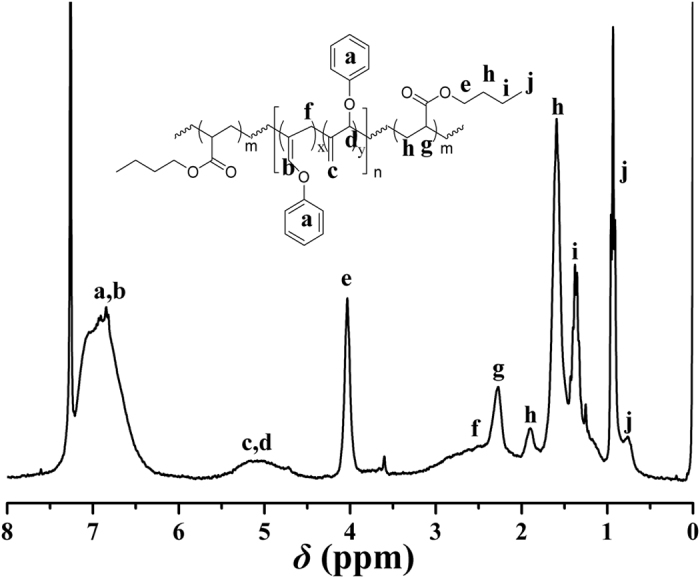
^1^H NMR spectrum of PBA-*b*-PPOA-*b*-PBA **4** in CDCl_3_.

The chemical structure of PBA-*b*-PPOA-*b*-PBA **4** triblock copolymer was examined by ^1^H NMR. Figure 8 exhibits ^1^H NMR spectrum of PBA-*b*-PPOA-*b*-PBA **4** triblock copolymer, which displayed all typical proton resonance signals of PPOA and PBA blocks. The peaks at 0.76, 0.93 (‘j’), 1.37 (‘i’), 1.59, 1.89 (‘h’), 2.27 (‘g’), and 4.05 (‘e’) were ascribed to all 12 protons of BA repeated unit, i.e. C*H*_2_C*H*CO_2_C*H*_2_C*H*_2_C*H*_2_C*H*_3_, respectively. Meanwhile, the double bonds in PPOA segment kept inert during ATRP of BA since their proton resonance signals around 5.10 ppm were still visible after the polymerization of BA monomer. Thus, we can conclude from the aforementioned evidences that double-bond- containing triblock copolymers comprising polyallene-based block and polyacrylate-based PBA segment, i.e. PBA-*b*-PPOA-*b*-PBA **4** triblock copolymers, were successfully constructed via Br-PPOA-Br **2** initiated ATRP of BA.

### The stability of PMMA-*b*-PPOA-*b*-PMMA and PBA-*b*-PPOA-*b*-PBA triblock copolymers

Since most of crosslinking and degradation of common polymers under atmosphere follow the radical mechanism and the as-prepared polyallene-based triblock copolymers bear labile double bonds in PPOA block, we therefore examined the stability of Br-PPOA-Br **2** macroinitiator and PMMA-*b*-PPOA-*b*-PMMA **3d** triblock copolymer under radical circumstance.

**Figure 9:**
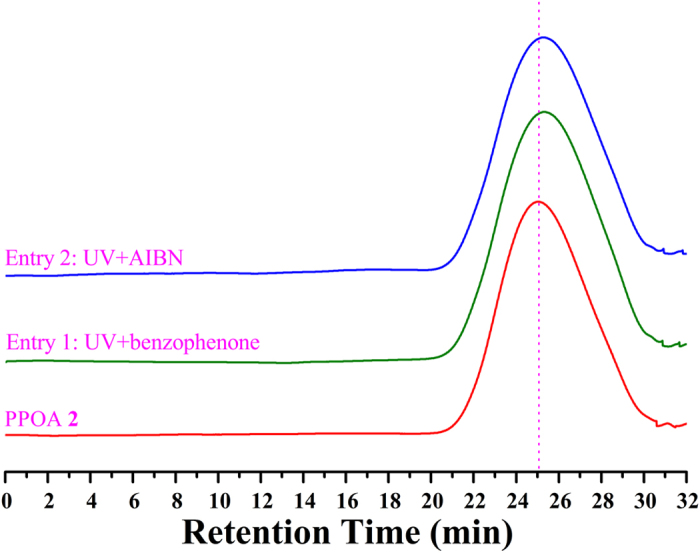
GPC traces of Br-PPOA-Br **2** before and after UV irradiation in THF.

For Br-PPOA-Br **2** macroinitiator, it was exposed to UV irradiation plus benzophenone (photo-sensitizer) or AIBN (initiator for free radical polymerization) at ambient temperature (Table S1), respectively. As shown in Fig. 9, the peak value and shape of elution peak in GPC retention curves after UV irradiation, i.e. molecular weight and polydispersity, were almost same with those in original GPC curve, which meant Br-PPOA-Br **2** macroinitiator did not degrade or cross-link during the test.

**Figure 10:**
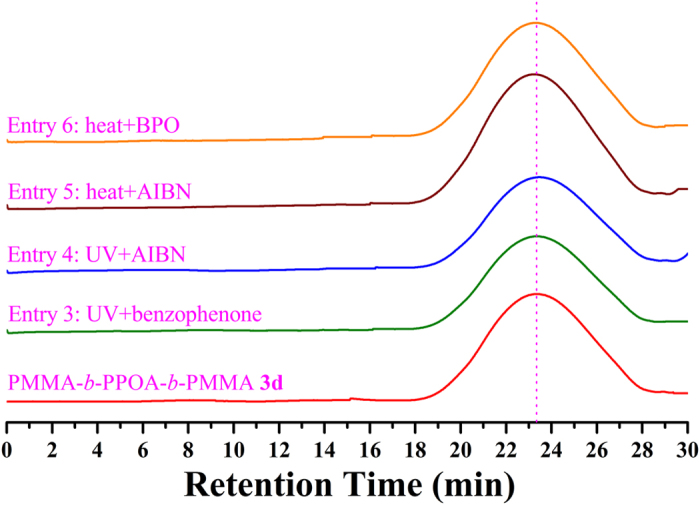
GPC traces of PMMA-*b*-PPOA-*b*-PMMA **3d** before and after heat initiation and UV irradiation in THF.

More importantly, PMMA-*b*-PPOA-*b*-PMMA **3d** triblock copolymer was exposed to UV irradiation same as the macroinitiator; furthermore, it was heated with AIBN or BPO at 80 °C (above the decomposition temperature of AIBN and BPO) as listed in Table S1. Excitingly, it can be seen from Fig. 10 that the peak value and shape of elution peak in GPC retention curves have no obvious change after the stability experiment, which clearly confirmed the double-bond-containing polyallene-based triblock polymers are stable under radical circumstance, either with UV irradiation or with heat initiation. This point implied that double bonds in polyallene-based copolymer are stable under common radical surrounding, which may faciliate the potential further application.

## Conclusion

We have displayed the detailed synthesis of PMMA-*b*-PPOA-*b*-PMMA and PBA-*b*-PPOA-*b*-PBA triblock copolymers by the combination of free radical polymerization, ATRP and the site transformation strategy, employing a bifunctional initiator possessing azo and Br-containing ATRP initiating groups as starting material. Polyallene-based macroinitiator was prepared by free radical polymerization of phenoxyallene initiated by the bifunctional initiator, which contains two ATRP initiating groups at both ends evidenced by elemental analysis and GPC/MALS. The target triblock copolymers were obtained by ATRP of MMA and BA initiated by the macroinitiator, respectively; and the polymerization process showed first order kinetics. This class of triblock copolymer is the first report of block copolymer via allene derivative and (meth)acrylate. The application of these double-bond-containing triblock copolymers is being explored in our group since they showed the exciting stability under radical circumstance, either with UV irradiation or with heat.

It is clear that the development of the as-prepared bifunctional initiator will make a significant contribution to the synthesis of polyallene-based block copolymers because PMMA and PBA segments in the current case can be easily extended to various hydrophilic and hydrophobic polymers for yielding diverse hydrophobic and amphiphilic block copolymers bearing double-bond-containing polyallene block.

## Additional Information

**How to cite this article:** Ding, A. *et al*. Double-bond-containing polyallene-based triblock copolymers via phenoxyallene and (meth)acrylate. *Sci. Rep.* **7**, 43706; doi: 10.1038/srep43706 (2017).

**Publisher's note:** Springer Nature remains neutral with regard to jurisdictional claims in published maps and institutional affiliations.

## Supplementary Material

Supporting Information

## Figures and Tables

**Table 1:** Bulk Polymerization of MMA^a^.

Entry	Time (h)	Conv.^b^ (%)	*M*_n_^c^ (g/mol)	*M*_w_/*M*_n_^c^
3a	0.5	1.0	10,100	1.66
3b	1.0	3.5	12,600	1.93
3c	1.5	5.5	15,500	1.98
3d	2.0	7.5	16,600	2.22

^a^Initiated by Br-PPOA-Br **2** macroinitiator (*M*_n,GPC/MALS_ = 9,200 g/mol, *M*_w_/*M*_n_ = 1.95), [MMA]:[Br group]:[CuBr]:[dHbpy] = 200:1:1:1, polymerization temperature: 50 °C.

^b^Determined by GC.

^c^Measured by GPC in THF at 35 °C.

**Table 2:** Bulk Polymerization of BA^a^.

Entry	Time (h)	Conv.^b^ (%)	*M*_n_^c^ (g/mol)	*M*_w_/*M*_n_^c^
4a	6.0	2.5	11,400	2.06
4b	8.0	4.1	13,600	2.11
4c	10.0	6.1	14,600	2.19

^a^Initiated by Br-PPOA-Br **2** macroinitiator (*M*_n,GPC/MALS_ = 9,200 g/mol, *M*_w_/*M*_n_ = 1.95), [BA]:[Br group]:[CuBr]:[PMDETA] = 200:1:1:1, polymerization temperature: 80 °C.

^b^Determined by GC.

^c^Measured by GPC in THF at 35 °C.

## References

[b1] McGrath M. P., Sall E. D. & Tremont S. J. Functionalization of polymers by metal-mediated processes. Chem. Rev. 95, 381–398 (1995).

[b2] Endo T. & Tomita I. Novel polymerization methods for allene derivatives. Prog. Polym. Sci. 22, 565–600 (1997).

[b3] Tomita I., Kondo Y., Takagi K. & Endo T. A novel living coordination polymerization of methoxyallene by π-allylnickel catalysyt. Macromolecules 27, 4413–4414 (1994).

[b4] Takagi K., Tomita I. & Endo T. A novel living coordination polymerization of phenylallene derivatives by π-allylnickel catalyst. Macromolecules 30, 7386–7390 (1997).

[b5] Endo T., Takagi K. & Tomita I. Design and synthesis of polymerizable cumulated double bond system. living coordination polymerization of alkylallenes by π-allylnickel catalyst. Tetrahedron 53, 15187–15196 (1997).

[b6] Takagi K., Tomita I. & Endo T. Living coordination polymerization of *N*- allenylamides by π-allylnickel catalysts. Macromolecules 31, 6741–6747 (1998).

[b7] Taguchi M., Tomita I. & Endo T. Living coordination polymerization of allene derivatives bearing hydroxy groups by π-allylnickel catalyst. Angew. Chem. Int. Ed. 39, 3667–3669 (2000). 10.1002/1521-3773(20001016)39:20<3667::aid-anie3667>3.0.co;2-g 11091433

[b8] Takagi K. & Tomita I. Synthesis of poly(alcohol)s by hydroboration/oxidation of poly(methylallene) prepared by π-allylnickel-catalyzed living coordination polymerization. Polym. Bull. 55, 251–258 (2005).

[b9] Kyohei M. & Tomita I. π-Allylnickel-catalyzed living coordination polymerization of allene having homochiral phenylcarbamoyloxy-substituted binaphthyl function. Macromolecules 39, 6336–6340 (2006).

[b10] Takagi K., Tomita I. & Endo T. Single-feed one-step block copolymerization of *n*-octyloxyallene with phenylallene using π-allylnickel as initiator. Chem. Commun. 681–682 (1998).

[b11] Taguchi M., Tomita I. & Endo T. Living coordination polymerization of allene (1,2-propadiene) by π-allylnickel catalyst and selective hydrosilylation reaction of polymers having polyallene units. Macromol. Chem. Phys. 201, 2322–2327 (2000).

[b12] Taguchi M., Tomita I., Yoshida Y. & Endo T. Block copolymerization of allene derivatives with 1,3-butadiene by living coordination polymerization with π-allylnickel catalyst. J. Polym. Sci. Polym. Chem. 37, 3916–3921 (1999).

[b13] Tomita I., Taguchi M., Takagi K. & Endo T. Block copolymerization of allene derivatives with isocyanides by the coordination polymerization with π-allylnickel catalyst. J. Polym. Sci. Polym. Chem. 35, 431–437 (1997).

[b14] Hu Y. Y. . Multiple stimuli-responsive and white-light emission of one-pot synthesized block copolymers containing poly(3-hexylthiophene) and poly(triethyl glycol allene) segments. Macromolecules 48, 5204–5212 (2015).

[b15] Yu Z. P. . Polyallene-*block*- polythiophene-*block*-polyallene copolymers: one-pot synthesis, helical assembly, and multiresponsiveness. Macromolecules 49, 1180–1190 (2016).

[b16] Yokozawa T., Ito N. & Endo T. Radical polymerization behavior of phenylallene. synthesis of functional polymer containing styryl moiety on the backbone. Chem. Lett. 17, 1955–1958 (1988).

[b17] Bhargava P. . Self-assembled polystyrene-*block*-poly(ethylene oxide) micelle morphologies in solution. Macromolecules 39, 4880–4888 (2006).

[b18] Walther A. . Multiple morphologies, phase transitions, and cross-linking of crew-cut aggregates of polybutadiene-*block*-poly(2-vinylpyridine) diblock copolymers. Macromolecules 41, 3254–3260 (2008).

[b19] Du V. A., Qiu H. B., Winnik M. A., Whittell G. R. & Manners I. Synthesis and solution self-assembly of polyisoprene-*block*-poly(ferrocenylmethylsilane): a diblock copolymer with an atactic but semicrystalline core-forming metalloblock. Macromol. Chem. Phys. 217, 1671–1682 (2016).

[b20] Banerjee A., Maji T., Paira T. K. & Mandal T. K. Diblock copolymers with miscible blocks via one-pot sequential cationic polymerization and their block-length-dependent vesicular aggregation. Macromol. Chem. Phys. 215, 440–451 (2014).

[b21] Tamotsu Hashimoto T., Imaeda T., Irie S., Urushisaki M. & Sakaguchi T. Synthesis of poly(vinyl ether)-based, ABA triblock-type thermoplastic elastomers with functionalized soft segments and their gas permeability. J. Polym. Sci. Polym. Chem. 53, 1114–1124 (2015).

[b22] Elladiou M. & Patrickios C. S. ABC triblock terpolymers with orthogonally deprotectable blocks: synthesis, characterization, and deprotection. Macromolecules 48, 7503–7512 (2015).

[b23] Uchiike C. . Evolution of iron catalysts for effective living radical polymerization: design of phosphine/halogen ligands in FeX_2_(PR_3_)_2_. Macromolecules 40, 8658–8662 (2007).

[b24] Mueller L., Jakubowski W., Tang W. & Matyjaszewski K. Successful chain extension of polyacrylate and polystyrene macroinitiators with methacrylates in an ARGET and ICAR ATRP. Macromolecules 40, 6464–6472 (2007).

[b25] Lligadas G., Ladislaw J. S., Guliashvili T. & Percec V. Functionally terminated poly(methyl acrylate) by SET-LRP initiated with CHBr_3_ and CHI_3_. J. Polym. Sci. Polym. Chem. 46, 278–288 (2008).

[b26] Jiang B. Y. . Phase behavior of alkyne-functionalized styrenic block copolymer/cobalt carbonyl adducts and *in situ* formation of magnetic nanoparticles by thermolysis. Macromolecules 49, 853–865 (2016).

[b27] Yagci Y. & Tasdelen A. M. Mechanistic transformations involving living and controlled/living polymerization methods. Prog. Polym. Sci. 31, 1133–1170 (2006).

[b28] Bernaerts K. V., Willet N., Van Camp W., Jerome R. & Du Prez F. E. pH-Responsive diblock copolymers prepared by the dual initiator strategy. Macromolecules 39, 3760–3769 (2006).

[b29] Villarroya S., Zhou J. X., Duxbury C. J., Heise A. & Howdle S. M. Synthesis of semifluorinated block copolymers containing poly(ε-caprolactone) by the combination of ATRP and enzymatic ROP in scCO_2_. Macromolecules 39, 633–640 (2006).

[b30] Triftaridou A. I., Loizou E. & Patrickios C. S. Synthesis and characterization of amphiphilic cationic symmetrical ABCBA pentablock terpolymer networks: effect of hydrophobic content. J. Polym. Sci. Polym. Chem. 46, 4420–4432 (2008).

[b31] Li H. . Synthesis of photocleavable poly(methyl methacrylate-block- D-lactide) via atom-transfer radical polymerization and ring-opening polymerization. J. Polym. Sci. Polym. Chem. 51, 4309–4316 (2013).

[b32] Fan X. S. . Novel linear-dendritic-like amphiphilic copolymers: synthesis and self-assembly characteristics. Polym. Chem., 5, 4069–4075 (2014).

[b33] Le D., Phan T. N. T., Autissier L., Charles L. & Gigmes D. A well-defined block copolymer synthesis via living cationic polymerization and nitroxide- mediated polymerization using carboxylic acid-based alkoxyamines as a dual initiator. Polym. Chem., 7, 1659–1667 (2016).

[b34] Wang J. S. & Matyjaszewski K. Controlled/“living” radical polymerization. atom transfer radical polymerization in the presence of transition-metal complexes. J. Am. Chem. Soc. 117, 5614–5615 (1995).

[b35] Percec V. & Barboiu B. “Living” radical polymerization of styrene initiated by arenesulfonyl chlorides and CuI(bpy)_n_Cl. Macromolecules 28, 7970–7972 (1995).

[b36] Kato M., Kamigaito M., Sawamoto M. & Higashimura T. Polymerization of methyl methacrylate with the carbon tetrachloride/dichlorotris(triphenyl- phosphine)ruthenium(II)/methylaluminum bis(2,6-di-*tert*-butylphenoxide) initiating system: possibility of living radical polymerization. Macromolecules 28, 1721–1723 (1995).

[b37] Williams V. A., Ribelli T. G., Chmielarz P., Park S. & Matyjaszewski K. A silver bullet: elemental silver as an efficient reducing agent for atom transfer radical polymerization of acrylates. J. Am. Chem. Soc. 137, 1428–1431 (2015). 25599253 10.1021/ja512519j

[b38] Wang F. . Synthesis of block copolymers containing polybutadiene segments by combination of coordinative chain transfer polymerization, ring-opening polymerization, and atom transfer radical polymerization. Macromol. Chem. Phys. 216, 321–328 (2015).

[b39] Shin S., Moon S., Seo M. & Kim S. Y. Synthesis of coil-comb block copolymers containing polystyrene coil and poly(methyl methacrylate) side chains via atom transfer radical polymerization. J. Polym. Sci. Polym. Chem. 54, 2971–2983 (2016).

[b40] Percec V. . Ultrafast synthesis of ultrahigh molar mass polymers by metal-catalyzed living radical polymerization of acrylates, methacrylates, and vinyl chloride mediated by SET at 25 °C. J. Am. Chem. Soc. 128, 14156–14165 (2006). 17061900 10.1021/ja065484z

[b41] Nguyen N. H., Rosen B. M., Lligadas G. & Percec V. Surface-dependent kinetics of Cu(0)-wire-catalyzed single-electron transfer living radical polymerization of methyl acrylate in DMSO at 25°C. Macromolecules 42, 2379–2386 (2009).

[b42] Anastasaki A. . High molecular weight block copolymers by sequential monomer addition via Cu(0)-mediated living radical polymerization (SET-LRP): an optimized approach. ACS Macro Lett. 2, 896–900 (2013). 10.1021/mz4004198 35607010

[b43] Zhang Q., Wilson P., Anastasaki A., McHale R. & Haddleton D. M. Synthesis and aggregation of double hydrophilic diblock glycopolymers via aqueous SET-LRP. ACS Macro Lett. 3, 491–495 (2014). 10.1021/mz5001724 35590789

[b44] Simula A. . Synthesis of well-defined α,ω-telechelic multiblock copolymers in aqueous medium: *in situ* generation of α,ω-diols. Polym. Chem. 6, 2226–2233 (2015).

[b45] Aksakal R., Resmini M. & Becer C. R. Pentablock star shaped polymers in less than 90 minutes via aqueous SET-LRP. Polym. Chem. 7, 171–175 (2016).

[b46] Moad G., Rizzardo E. & Thang S. H. Toward Living Radical Polymerization. Acc. Chem. Res. 41, 1133–1142 (2008). 18700787 10.1021/ar800075n

[b47] Moad G., Rizzardo E. & Thang S. H. Radical addition–fragmentation chemistry in polymer synthesis. Polymer 49, 1079–1131 (2008).

[b48] Zhang X. H., Peng D., Lu G. L., Gu L. N. & Huang X. Y. A novel graft copolymer containing polyallene backbone and poly(*tert*-butyl acrylate) side chains. J. Polym. Sci. Polym. Chem. 44, 6888–6893 (2006).

[b49] Zhang X. H. . Synthesis of polyallene-based graft copolymer via 6-methyl- 1,2-heptadien-4-ol and styrene. J. Polym. Sci. Polym. Chem. 45, 5509–5517 (2007).

[b50] Zhang X. H. . Polyallene-based graft copolymer via 6-methyl-1,2-heptadien- 4-ol and methyl methacrylate. Polymer 48, 5507–5513 (2007).

[b51] Matyjaszewski K. & Xia J. H. Atom transfer radical polymerization. Chem. Rev. 101, 2921–2990 (2001). 11749397 10.1021/cr940534g

