# Supplementary material for: Double-bond-containing polyallene-based triblock copolymers via phenoxyallene and (meth)acrylate
Source: Sci Rep. 2017 Mar 2;7:43706. doi: 10.1038/srep43706 (PMC5333076; doi:10.1038/srep43706)
Supplement: Supporting Information [file srep43706-s1.doc]

**Supporting information for**

**Double-bond-containing polyallene-based triblock copolymers via phenoxyallene and (meth)acrylate**

*Aishun Ding*,*^1^*^,2,#^ *Guolin Lu*,*^2^*^,#^ *Hao Guo*,*^1^*,* *Xiaoyu Huang^2^*,***

^1^ Department of Chemistry, Fudan University, 220 Handan Road, Shanghai 200433, People’s Republic of China

^2^ Key Laboratory of Synthetic and Self-Assembly Chemistry for Organic Functional Molecules, Shanghai Institute of Organic Chemistry, Chinese Academy of Sciences, 345 Lingling Road, Shanghai 200032, People’s Republic of China

**Experimental Section**

**Materials**

Methyl methacrylate (MMA, Aldrich, 99%) and *n*-butyl acrylate (BA, Aldrich, 99%) were washed with 5% aqueous NaOH solution to remove the inhibitor, then washed with water, dried over CaCl_2_ and distilled twice *in vacuo* from CaH_2_ prior to use. Copper(I) bromide (CuBr, Aldrich, 98%) was purified by stirring overnight over CH_3_CO_2_H at room temperature, followed by washing the solid with ethanol, acetone, and diethyl ether prior to drying *in vacuo* at 40^o^C for 1 day. Triethylamine (Aldrich, 99.5%) was dried over KOH for several days followed by distilling from CaH_2_ under Ar prior to use. Benzoyl peroxide (BPO, Aldrich, 97%) was recrystallized from chloroform and methanol at room temperature. 2,2’-Azobis(isobutyronitrile) (AIBN, Aldrich, 98%) was recrystallized from anhydrous ethanol. Tetrahydrofuran (THF, Aldrich, 99%) and toluene (Aldrich, 99.5%) were dried over CaH_2_ for a week and distilled from sodium and benzophenone under Ar prior to use. 2-Bromopropionyl chloride (2-BPC, Aldrich), hydroquinone (Aldrich, 99%), benzophenone (BP, Aldrich, 99%), thionyl chloride (Aldrich 99%), *N*,*N*,*N*’,*N*’,*N*’’-pentamethyldiethylenetriamine (PMDETA, Aldrich, 99%), and 4,4’-azobis(4-cyanopentanoic acid) (Acros, 97%) were used as received. Diheptyl-2,2'-bipyridine (dHbpy)^1^ and phenoxyallene (POA)^2^ were synthesized according to previous literatures.

**Measurements**

FT-IR spectra were recorded on a Nicolet AVATAR-360 FTIR spectrophotometer with a resolution of 4 cm^-1^. All ^1^H (500 MHz) and ^13^C (125 MHz) NMR analyses were performed on a Bruker Avance 500 spectrometer in CDCl_3_, TMS (^1^H NMR) and CDCl_3_ (^13^C NMR) were used as internal standards. Elemental analysis was carried out on a Carlo-Erba1106 system. Bromine content was determined by the titration with Hg(NO_3_)_2_. Conversions of MMA and BA were determined by gas chromatography (GC) using a HP 6890 system with an SE-54 column. Relative molecular weights and molecular weight distributions were measured by conventional gel permeation chromatography (GPC) system equipped with a Waters 1515 Isocratic HPLC pump, a Waters 2414 refractive index detector, and a set of Waters Styragel columns (HR3 (500-30,000), HR4 (5,000-600,000), and HR5 (50,000-4,000,000), 7.8×300 mm, particle size: 5 μm). GPC measurements were carried out at 35^o^C using THF as eluent with a flow rate of 1.0 mL/min. The system was calibrated with linear polystyrene standards. Absolute molecular weight of the macroinitiator was determined by GPC equipped with a multiangle light scattering detector (GPC/MALS), THF was used as the eluent with a flow rate of 1.0 mL/min, detectors: Wyatt Optilab rEX refractive index detector and Wyatt DAWN HELEOS 18-angle light scattering detector with a 50 mW solid-state laser operating at 658 nm.

**Synthesis of Azo-ATRP Bifunctional Initiator**

Bifunctional initiator **1** was synthesized in three steps using commercially available 2-BPC, hydroquinone, and 4,4’-azobis(4-cyanopentanoic acid) as starting materials (Scheme S1).


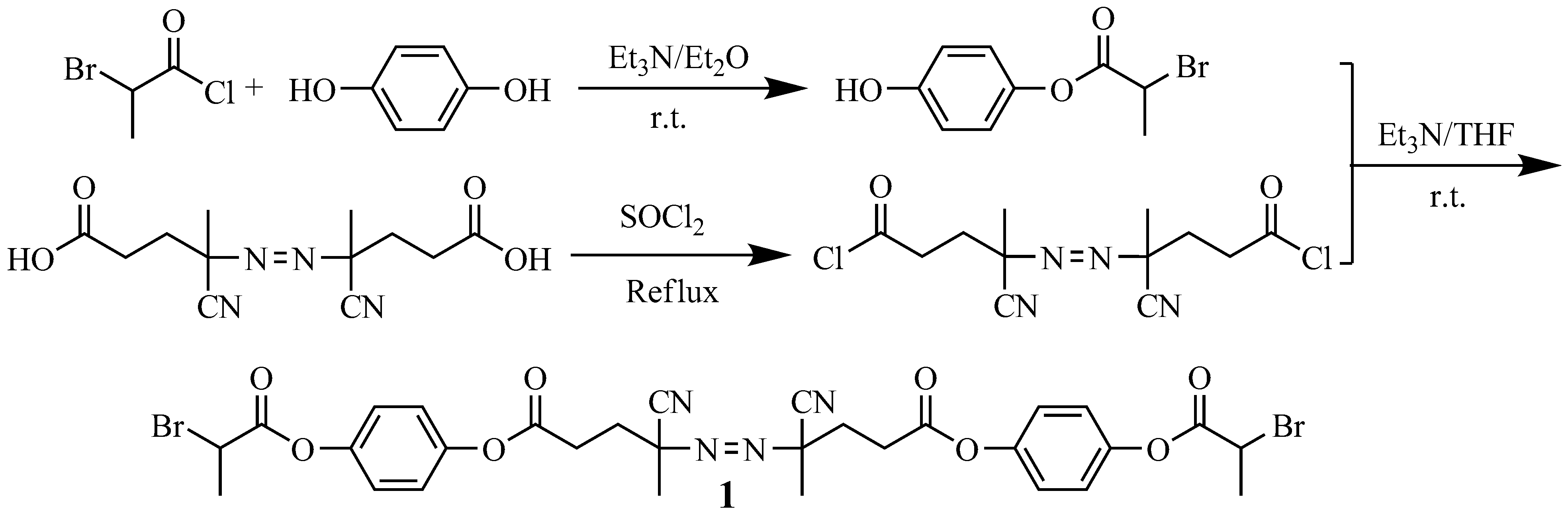


**Scheme S1.** Synthesis of Azo-ATRP Bifunctional Initiator **1**

Hydroquinone (2.30 g, 20.91 mmol), diethyl ether (150 mL) and triethylamine (4 mL) were added to a 250 mL three-neck flask followed by adding 2-BPC (2 mL, 19.83 mmol) dropwise. After the reaction was complete monitored by thin layer chromatography (TLC), the mixture was filtered and the filtrate was concentrated. The purified 4-hydroxyphenyl 2-bromopropanoate (3.43 g, 70.5%) was obtained by silica column chromatography.

The mixture of 4,4’-azobis(4-cyanopentanoic acid) (4.2 g, 15 mmol) and thionyl chloride (30 mL) was refluxed at 75^o^C for 1 h. After the unreacted thionyl chloride was removed by pumping, the residue (yellow solid) was dissolved in 50 mL of THF and this solution was then added dropwise to the mixture of 4-hydroxyphenyl 2-bromopropanoate (7.92 g) and triethylamine (4.2 mL) in 100 mL of THF. After the reaction was complete monitored by TLC, the mixture was filtered and the filtrate was concentrated followed by silica column chromatography and recrystallization from methanol to provide the desired azo-ATRP bifunctional initiator **1** (white solid) with a yield of 31.5% (3.47 g). Anal. Calcd. for C_30_H_30_Br_2_N_4_O_8_: Calcd: C, 49.06%; H, 4.12%; N, 7.63%; Br, 21.76%. Found: C,49.40%; H, 4.02%; N, 7.36%; Br, 21.49%. FT-IR: *ν* (cm^-1^): 3116, 3075, 2995, 2925, 2237 (C≡N), 1756 (C=O), 1502, 1445, 1339, 1182, 1135, 930, 897, 840, 523 (C-Br). ^1^H NMR: *δ* (ppm): 1.75 (6H, C*H*_3_C(CN)CH_2_CH_2_CO_2_), 1.95 (6H, C*H*_3_CHBr), 2.61 (8H, CH_3_C(CN)C*H*_2_C*H*_2_CO_2_), 4.59 (2H, CH_3_C*H*Br), 7.13 (8H, C_6_*H*_4_). ^13^C NMR: *δ* (ppm): 21.4, 24.0, 29.1, 33.0, 39.4, 117.4, 122.2, 148.0, 168.5, 169.7.

**Free Radical Homopolymerization of Phenoxyallene**

Bifunctional initiator **1** (0.3694 g, 0.50 mmol) was first added to a 100 mL Schlenk flask (flame-dried *in vacuo* prior to use) sealed with a rubber septum for degassing and kept under Ar. Next, POA (12 mL, 101.8 mmol) and toluene (12 mL) were introduced via a gastight syringe. The solution was degassed by three cycles of freezing-pumping-thawing followed by immersing the flask into an oil bath set at 75^o^C to start the polymerization. The polymerization was terminated by immersing the flask into liquid N_2_ after 24 h. The reaction mixture was diluted by THF and precipitated into *n*-hexane. After repeated purification by dissolving in THF and precipitating in *n*-hexane, 6.3852 g of white powder, poly(phenoxyallene) (PPOA) **2** homopolymer, was obtained after drying *in vacuo* overnight. GPC: *M*_n,GPC_ = 9,400 g/mol, *M*_w_/*M*_n_ = 1.98. GPC/MALS: *M*_n,GPC/MALS_ = 9,200 g/mol, *M*_w_/*M*_n_ = 1.95. Element analysis: Br% = 1.72%. FT-IR: *ν* (cm^-1^): 3070, 3039, 2952, 2926, 1761 (C=O), 1675, 1643 (C=C), 1595, 1491, 1456, 1337, 1290, 1227, 1171, 1135, 1030, 892, 752, 691. ^1^H NMR: *δ* (ppm): 1.61 (6H, C*H*_3_CCN), 1.93 (6H, C*H*_3_CHBr), 2.55 (2H×x, =C-C*H*_2_), 4.57 (2H, CH_3_C*H*Br), 5.00 (1H×y, CH_2_=CC*H*O), 5.21 (2H×y, C*H*_2_=CCHO), 6.85, 6.92, 7.01, 7.16 (1H×x, C=C*H*OC_6_H_5_ and 5H, C_6_*H*_5_). ^13^C NMR: *δ* (ppm): 19.4, 21.3, 25.5, 28.9, 30.4, 33.5, 35.9, 39.4, 72.6, 112.5, 115.7, 116.6, 120.8, 122.7, 141.8, 158.3, 169.0.

**Block Copolymerization of Methyl Methacrylate**

PPOA **2** macroinitiator (*M*_n,GPC/MALS_ = 9,200 g/mol, *M*_w_/*M*_n_ = 1.95), CuBr, and dHbpy were first added to a 25 mL Schlenk flask (flame-dried *in vacuo* prior to use) sealed with a rubber septum for degassing and kept under Ar. MMA monomer was charged via a gastight syringe and the flask was degassed by three cycles of freezing-pumping-thawing followed by immersing the flask into an oil bath set at 50^o^C. The polymerization was quenched by putting the flask into liquid N_2_ after certain time. The reaction mixture was diluted by THF and passed through an alumina column to remove the residual copper catalyst. The solution was concentrated and precipitated into *n*-hexane. After repeated purification by dissolving in THF and precipitating in *n*-hexane, the crude product was dried *in vacuo* overnight to afford the final product (white powder), PMMA-*b*-PPOA-*b*-PMMA **3** triblock copolymer. FT-IR: *ν* (cm^-1^): 3062, 3034, 2953, 2925, 1731 (C=O), 1595, 1491, 1455, 1229, 1169, 1152, 752, 690. ^1^H NMR: *δ* (ppm): 0.84, 1.02, 1.23 (3H×m, CH_2_CC*H*_3_), 1.61, 1.81 (2H×m, C*H*_2_CCH_3_), 2.57 (2H×x, =C-C*H*_2_), 3.59 (3H×m, CO_2_C*H*_3_), 5.00 (1H×y, CH_2_=CC*H*O), 5.21 (2H×y, C*H*_2_=CCHO), 6.85, 6.91, 7.06 (1H×x, C=C*H*OC_6_H_5_ and 5H, C_6_*H*_5_).

For kinetics investigation, the first sample taken as a time = 0 data point was obtained by withdrawing 0.50 mL of solution from the flask using a purged syringe and adding to 2.00 mL of THF. The flask was immersed into an oil bath set at 50^o^C. At every time interval, 0.50 mL sample solution was taken and added to 2.00 mL THF. Every sample solution in THF taken at different time was injected into GC to determine the conversion of MMA compared with the time = 0 data point.

**Block Copolymerization of Butyl Acrylate**

PPOA **2** macroinitiator (*M*_n,GPC/MALS_ = 9,200 g/mol, *M*_w_/*M*_n_ = 1.95), CuBr, and PMDETA were first added to a 25 mL Schlenk flask (flame-dried *in vacuo* prior to use) sealed with a rubber septum for degassing and kept under Ar. BA monomer was charged via a gastight syringe and the flask was degassed by three cycles of freezing-pumping-thawing followed by immersing the flask into an oil bath set at 80^o^C. The polymerization was quenched by putting the flask into liquid N_2_ after certain time. The reaction mixture was diluted by THF and passed through an alumina column to remove the residual copper catalyst. The solution was concentrated and precipitated into *n*-hexane. After repeated purification by dissolving in THF and precipitating in *n*-hexane, the crude product was dried *in vacuo* overnight to afford the final product (white powder), PBA-*b*-PPOA-*b*-PBA **4** triblock copolymer. FT-IR: *ν* (cm^-1^): 3058, 3043, 2958, 2926, 1735 (C=O), 1596, 1492, 1459, 1225, 1168, 1029, 752, 691. ^1^H NMR: *δ* (ppm): 0.76, 0.93 (3H×m, CO_2_CH_2_CH_2_CH_2_C*H*_3_), 1.37 (2H×m, CO_2_CH_2_CH_2_C*H*_2_CH_3_), 1.59, 1.89 (2H×m, CO_2_CH_2_C*H*_2_CH_2_CH_3_ and 2H×m, C*H*_2_CH), 2.27 (1H×m, CH_2_C*H*), 2.52 (2H×x, =C-C*H*_2_), 4.03 (2H×m, CO_2_C*H*_2_CH_2_CH_2_CH_3_), 5.08 (3H×y, C*H*_2_=CC*H*O), 6.85, 6.92, 7.06 (1H×x, C=C*H*OC_6_H_5_ and 5H, C_6_*H*_5_).

For kinetics investigation, the first sample taken as a time = 0 data point was obtained by withdrawing 0.50 mL of solution from the flask using a purged syringe and adding to 2.00 mL of THF. The flask was immersed into an oil bath set at 80^o^C. At every time interval, 0.50 mL sample solution was taken and added to 2.00 mL THF. Every sample solution in THF taken at different time was injected into GC to determine the conversion of BA compared with the time = 0 data point.

**Stability Test**

The stability of Br-PPOA-Br **2** macroinitiator and PMMA-*b*-PPOA-*b*-PMMA **3** triblock copolymer were tested under different free radical and UV irradiation conditions as summarized in Table S1.

**Table S1.** Stability Test Conditions^a^

| Entry | Sample | Condition | Temperature (^o^C) | Time (h) |
| --- | --- | --- | --- | --- |
| 1 | **2** | UV+benzophenone | 25 | 0.5 |
| 2 | **2** | UV+AIBN | 25 | 0.5 |
| 3 | **3d** | UV+benzophenone | 25 | 0.5 |
| 4 | **3d** | UV+AIBN | 25 | 0.5 |
| 5 | **3d** | heat+AIBN | 80 | 8.0 |
| 6 | **3d** | heat+BPO | 80 | 8.0 |

^a^ The amount of AIBN, BPO, and benzophenone is 5 equiv, solvent: toluene.

**References**

1. Leduc, M. R.; Hawker, C. I.; Dao, J.; Frechet, J. M. J. *J. Am. Chem. Soc.* **1996**, *118*, 11111-11118.
2. Boerresen, S.; Crandall, J. K. *J. Org. Chem*. **1976,** *41*, 678-681.
